# C-Reactive Protein-to-Albumin Ratio as a Prognostic Marker in ICU Patients with Pre-Existing Hypertension and Diabetes

**DOI:** 10.3390/jcm15103683

**Published:** 2026-05-11

**Authors:** Sultan Almuntashiri, Eissa A. Jafari, Abdullah Alhumaid, Abdulaziz H. Alanazi, Moaddey Alfarhan, Abdulkarim Alshammari

**Affiliations:** 1 Department of Clinical Pharmacy, College of Pharmacy, University of Ha’il, Ha’il 55473, Saudi Arabia; s.almuntashiri@uoh.edu.sa (S.A.); aalhumaid@ufl.edu (A.A.); 2 Department of Pharmacy Practice, College of Pharmacy, Jazan University, Jazan 45142, Saudi Arabia; malfarhan@jazanu.edu.sa; 3 Department of Pharmacotherapy & Translational Research, College of Pharmacy, University of Florida, Gainesville, FL 32610, USA; 4 Department of Pharmacy Practice, College of Pharmacy, Northern Border University, Rafha 91431, Saudi Arabia; abdulaziz.alanazi4@nbu.edu.sa (A.H.A.); abdulkarim.alshammari@nbu.edu.sa (A.A.)

**Keywords:** hypertension, diabetes, C reactive protein to albumin ratio, intensive care unit, critical care, mortality, prognosis

## Abstract

**Background/Objective**: The C-reactive protein-to-albumin ratio (CAR) reflects both systemic inflammation and nutritional status and has been proposed as a prognostic marker in critical illness, yet its value in intensive care unit (ICU) patients with pre-existing hypertension is not well defined. **Methods**: This was a retrospective single-center study of 341 critically ill adults with pre-existing hypertension (June 2001–October 2012) from the Medical Information Mart for Intensive Care III database. CAR was calculated from the first C-reactive protein (CRP) and albumin measurements after ICU admission. Using adjusted Cox proportional hazard models, we examined the association of CAR with 30-day mortality in the overall hypertensive cohort, across hypertension groups, and in patients with coexisting diabetes. **Results**: Non-survivors had higher CAR than survivors (35.5 vs. 18.1, *p* = 0.008). CAR showed moderate discriminative ability in the overall hypertensive cohort (AUC = 0.637, 95% CI: 0.543–0.732), with better discrimination in patients with normal/elevated blood pressure (BP) (AUC = 0.748, 95% CI: 0.637–0.858) and a relatively higher AUC in the subgroup with diabetes and normal/elevated BP (0.833, 95% CI: 0.671–0.995). In univariable Cox analysis, high CAR was associated with increased 30-day mortality in the overall hypertensive cohort (HR: 3.02, 95% CI: 1.48–6.17, *p* = 0.0024), in patients with normal/elevated BP (HR: 8.90, 95% CI: 2.00–39.17, *p* = 0.0038), and in patients with diabetes and normal/elevated BP (HR: 10.00, 95% CI: 1.20–83.10, *p* = 0.0331). These associations remained significant after multivariable adjustment in the overall hypertensive cohort (adjusted HR: 3.01, 95% CI: 1.45–6.21, *p* = 0.0030), in patients with normal/elevated BP (adjusted HR: 10.12, 95% CI: 2.20–46.59, *p* = 0.0030), and in patients with diabetes and normal/elevated BP (adjusted: HR: 19.41, 95% CI: 1.37–275.28, *p* = 0.0284). **Conclusions**: These results suggest that CAR measured early after ICU admission may serve as a practical tool for mortality risk stratification in ICU patients with pre-existing hypertension, particularly those with diabetes.

## 1. Introduction

Critically ill patients admitted to the intensive care unit (ICU) remain at high risk for poor outcomes, including multi-organ failure, severe infections, hemodynamic instability, and ultimately short-term mortality [1]. Despite advances in supportive care, predicting outcomes in this population remains challenging. Early identification of patients at increased risk is essential for timely intervention, closer monitoring, and informed decision-making [2]. Simple biomarkers derived from routine laboratory tests that can predict severity or mortality are valuable, as they can be rapidly obtained, are widely available, and can be used to support clinical risk stratification or guide treatment strategies [3].

Arterial hypertension is one of the most common chronic diseases worldwide and is frequently present among ICU patients [4]. It is increasingly recognized as a condition associated with systemic inflammation, endothelial dysfunction, and vascular injury, all of which may contribute to poor outcomes during critical illness [5,6]. Diabetes mellitus, which often coexists with hypertension, shares many overlapping mechanisms, including chronic low-grade inflammation and metabolic stress [7]. Given the high prevalence of these two conditions occurring together, patients with both hypertension and diabetes may represent a particularly vulnerable subgroup. Evaluating prognostic markers in this population is crucial for improving both early risk assessment and clinical management strategies.

Among routinely available laboratory markers, C-reactive protein (CRP) reflects systemic inflammation and rises rapidly in response to acute triggers, while albumin reflects nutritional status, physiological reserve, and declines during systemic stress [8]. The ratio of CRP to albumin (CAR) combines these two markers and provides a more integrated reflection of inflammatory and nutritional status [8]. CAR has been studied as a prognostic marker in several disease states and in critically ill patients [9,10,11], but evidence in those with hypertension and diabetes remains very limited. The objective of the present study was to evaluate the prognostic significance of CAR measured shortly after ICU admission in patients with pre-existing hypertension. In particular, we aimed to assess its predictive value for 30-day mortality across hypertension groups and in those with coexisting diabetes.

## 2. Materials and Methods

### 2.1. Study Design and Data Source

We conducted a retrospective cohort study using the publicly available Medical Information Mart for Intensive Care III (MIMIC-III, version 1.4) database. MIMIC-III contains detailed clinical data from over 60,000 ICU admissions at the Beth Israel Deaconess Medical Center (Boston, MA, USA). The MIMIC-III database includes data collected between June 2001 and October 2012 [12]. Access to the database was approved through the required data use agreement and certification process [12,13].

### 2.2. Study Population and Cohort Selection

Patients aged ≥ 18 with a pre-existing diagnosis of hypertension were identified using International Classification of Diseases, Ninth Revision (ICD-9) codes (Table S1) from the MIMIC-III database. Due to the nature of the MIMIC-III database, which focuses on hospitalization and ICU data, detailed outpatient measurements and diagnostic procedures used to establish hypertension were not available. Therefore, the specific numerical criteria used for the original diagnosis of hypertension could not be directly determined from the available data. Diabetes mellitus was defined based on ICD-9 diagnostic codes indicating a pre-existing diagnosis prior to ICU admission (Table S1). Detailed diagnostic criteria for diabetes and laboratory confirmation were not available in the dataset. Due to the retrospective design of the study and the structure of the MIMIC-III database, the primary reason for ICU admission could not be determined with certainty. For patients with multiple ICU admissions, only the first ICU stay was included. We then restricted the cohort to those with both CRP and albumin measurements available after ICU admission, which allowed calculation of the CAR. Patients without available CRP or albumin measurements were excluded. The final study population consisted of 341 ICU patients with preexisting hypertension.

### 2.3. Variables

Baseline demographic variables included age and gender. Clinical data included blood pressure (BP) category on ICU admission and the presence of pre-existing diabetes. BP categories were defined using the first available BP measurements obtained after ICU admission and classified according to the American Heart Association and American College of Cardiology BP categories as normal BP, elevated BP, stage 1 BP, stage 2 BP, and hypertensive crisis [14,15]. Briefly, BP categories in the present study were classified as follows: normal BP (SBP < 120 mmHg and DBP < 80 mmHg), elevated BP (SBP 120–129 mmHg and DBP < 80 mmHg), stage 1 BP (SBP 130–139 mmHg or DBP 80–89 mmHg), Stage 2 BP (SBP ≥ 140 mmHg or DBP ≥ 90 mmHg), and hypertensive crisis (SBP ≥ 180 mmHg or DBP ≥ 120 mmHg). Normal and elevated BP were combined into a single category. Because BP groups were based on measurements obtained on ICU admission rather than in outpatient or ambulatory settings, these categories do not represent standard hypertension staging. Also, blood pressure values on ICU admission may vary widely depending on the underlying acute illness and may not necessarily reflect chronic hypertension status. Laboratory variables included CRP, serum albumin, white blood cell (WBC) count, and creatinine. Laboratory values were defined as the first available measurements obtained after ICU admission. The CAR was calculated as CRP (mg/L) divided by albumin (g/dL). The primary outcome was 30-day all-cause mortality, defined as death within 30 days of ICU admission based on the recorded date of death (DOD), irrespective of whether it occurred during hospitalization or after discharge. All study data, including mortality outcomes, were obtained from the MIMIC-III database.

### 2.4. Statistical Analysis

Continuous variables were summarized as mean ± standard deviation (SD) if normally distributed, or as median with interquartile range (IQR) if non-normally distributed. Categorical variables were presented as counts and percentages. Between-group comparisons were performed using the *t*-test or Mann–Whitney U test for continuous variables, as appropriate, and chi-square test or Fisher’s exact test for categorical variables. Receiver operating characteristic (ROC) curve analysis was used to assess the discriminative performance of CAR, and the area under the curve (AUC) was estimated to quantify its overall predictive ability; the optimal cutoff value was determined using the Youden index. Survival analyses were performed using Kaplan–Meier methods, with differences assessed by the log-rank test. To further evaluate whether CAR is independently associated with 30-day mortality, we performed univariable and multivariable Cox proportional hazards regression analyses. Covariates for multivariable adjustment were selected based on clinical relevance and data availability and included age, sex, diabetes, congestive heart failure, coronary artery disease, chronic obstructive pulmonary disease, chronic kidney disease, WBC, creatinine, and mechanical ventilation. The results of the Cox proportional hazard model were reported as hazard ratio (HR) with their corresponding 95% confidence interval (CI) and *p*-value. A two-sided *p*-value < 0.05 was considered statistically significant. All analyses were conducted using R version 4.5.1 (R Foundation for Statistical Computing, Vienna, Austria).

## 3. Results

### 3.1. Baseline Characteristics

The demographic, clinical, and laboratory characteristics of the 341 ICU patients with pre-existing hypertension stratified by 30-day survival are summarized in Table 1. The median age of non-survivors was significantly higher compared with survivors (72.0 vs. 64.1 years, *p* = 0.011). Inflammatory markers demonstrated significant differences between survivors and non-survivors. The non-survivors had higher CRP levels (89.9 vs. 52.7 mg/L, *p* = 0.012), lower serum albumin (2.78 vs. 2.98 g/dL, *p* = 0.038), and consequently higher CAR values (35.5 vs. 18.1, *p* = 0.008). Gender distribution, hypertension group, diabetes prevalence, WBC count, and creatinine did not differ significantly between groups.

### 3.2. Association of CAR with Mortality Across Hypertension Groups

**Figure 1:**
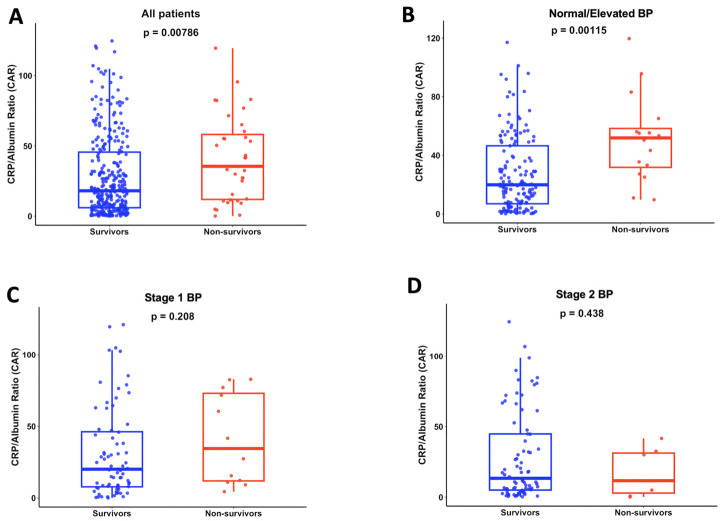
**Comparison of CAR between survivors and non-survivors.** (**A**) All patients: survivors (*n* = 306) vs. non-survivors (*n* = 35). (**B**) Normal/elevated BP: survivors (*n* = 149) vs. non-survivors (*n* = 16). (**C**) Stage 1 BP: survivors (*n* = 72) vs. non-survivors (*n* = 12). (**D**) Stage 2 BP: survivors (*n* = 78) vs. non-survivors (*n* = 7). BP was determined on ICU admission.

We assessed the prognostic value of CAR across different hypertension groups to determine whether its association with mortality varied by disease severity. CAR values were significantly higher in non-survivors compared with survivors in the overall hypertensive cohort (35.5 vs. 18.1, *p* = 0.008) (Figure 1A). A similar difference was observed in patients with normal/elevated BP (51.8 vs. 19.9, *p* = 0.0011) (Figure 1B). However, CAR did not differ significantly between survivors and non-survivors in stage 1 BP (*p* = 0.208) or stage 2 BP patients (*p* = 0.438) (Figure 1C,D).

### 3.3. Predictive Performance of CAR for Mortality Across Hypertension Groups

**Figure 2:**
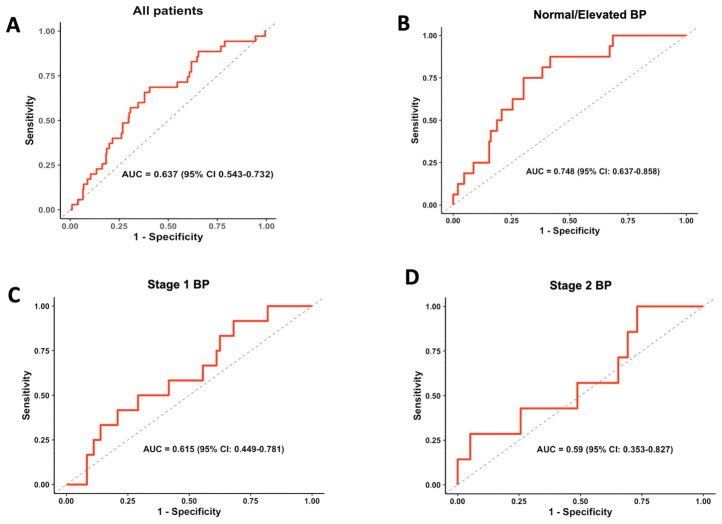
**ROC curves for the predictive value of CAR in hypertensive groups.** (**A**) All patients (*n* = 341), (**B**) Normal/elevated BP (*n* = 165), (**C**) Stage 1 BP (*n* = 84) and (**D**) Stage 2 BP (*n* = 85). BP was determined on ICU admission.

Next, we wanted to test whether CAR could serve as a predictive marker for mortality using ROC curve analysis across different hypertension groups. ROC curve analyses showed that CAR had moderate discriminative ability for predicting 30-day mortality in the overall hypertensive cohort (AUC = 0.637, 95% CI: 0.543–0.732) (Figure 2A). In patients with normal/elevated BP, CAR showed greater discriminative ability (AUC = 0.748, 95% CI: 0.637–0.858) with an optimal cutoff of 24.9, which provided a sensitivity of 87.5% and a specificity of 58.4% (Figure 2B). However, CAR showed limited predictive value in stage 1 and stage 2 BP patients (Figure 2C,D).

### 3.4. Prognostic Impact of CAR Using the Optimal Cutoff Across Hypertension Groups

**Figure 3:**
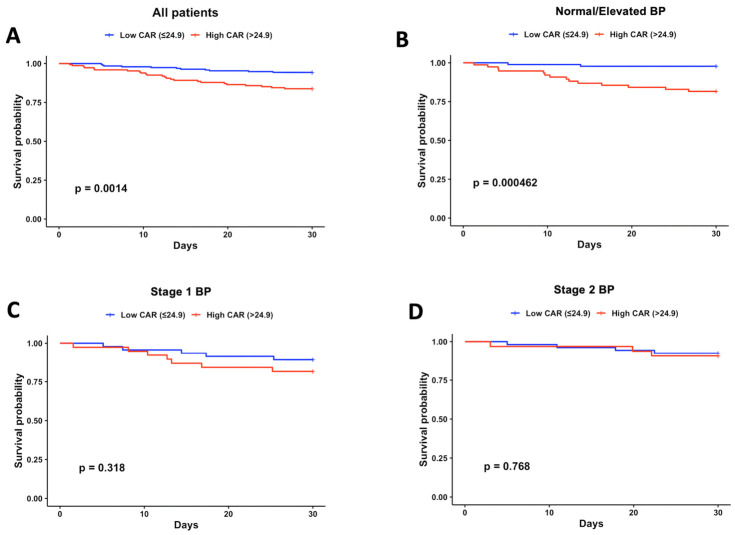
**Survival analysis of hypertension groups according to CAR cutoff level.** (**A**) All patients: low CAR (*n* = 193) vs. high CAR (*n* = 148). (**B**) Normal/elevated BP: low CAR (*n* = 89) vs. high CAR (*n* = 76). (**C**) Stage 1 BP: low CAR (*n* = 46) vs. high CAR (*n* = 38). (**D**) Stage 2 BP: low CAR (*n* = 53) vs. high CAR (*n* = 32). BP was determined on ICU admission.

After obtaining the optimal cutoff level, we evaluated the association between CAR and 30-day mortality across different hypertension groups. Among all patients, those with high CAR (>24.9) had a significantly higher 30-day mortality compared with low CAR (16.2% vs. 5.7%, *p* = 0.0014) (Figure 3A). This association was even more pronounced in the normal/elevated BP group (18.4% vs. 2.2%, *p* < 0.001) (Figure 3B). In contrast, no significant differences were observed in stage 1 BP (18.4% vs. 10.9%, *p* = 0.318) or stage 2 BP patients (9.4% vs. 7.5%, *p* = 0.768) (Figure 3C,D).

### 3.5. Prognostic Value of CAR in ICU Patients with Pre-Existing Hypertension and Diabetes

**Figure 4:**
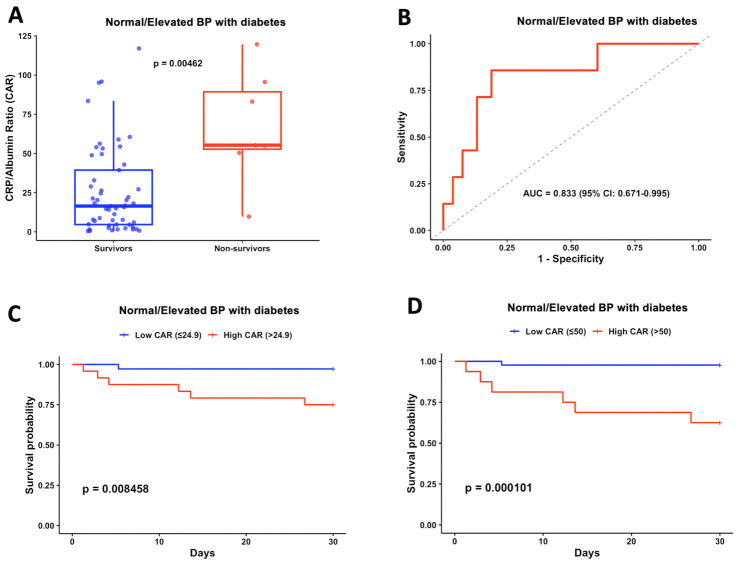
**Prognostic value of CAR in ICU patients with pre-existing hypertension and diabetes.** (**A**) Distribution of CAR in survivors (*n* = 53) and non-survivors (*n* = 7). (**B**) ROC curve in this subgroup (*n* = 60). (**C**) Survival analysis based on CAR cutoff 24.9 value: low CAR (*n* = 36) vs. high CAR (*n* = 24). (**D**) Survival analysis based on CAR cutoff 50: low CAR (*n* = 44) vs. high CAR (*n* = 16). BP was determined on ICU admission.

Since patients with normal/elevated BP showed the most pronounced association between CAR and mortality and given that diabetes is a frequent comorbidity in this population, we further examined this subgroup. In patients with normal/elevated BP and diabetes, non-survivors had significantly higher CAR values compared with survivors (55.3 vs. 16.4, *p* = 0.0046) (Figure 4A). ROC curve analysis showed a relatively high discriminative ability (AUC = 0.833, 95% CI: 0.671–0.995) with an optimal cutoff of approximately 50, providing a sensitivity of 85.7% and a specificity of 81.1% (Figure 4B). Using the original CAR threshold of 24.9, survival analysis showed that patients with high CAR (>24.9) experienced higher 30-day mortality than those with low CAR (25.0% vs. 2.8%, *p* = 0.008) (Figure 4C). As a sensitivity analysis, applying the ROC-derived cutoff of CAR 50 demonstrated an even more pronounced mortality gradient (37.5% vs. 2.3%, *p* < 0.001) (Figure 4D). These findings suggest that CAR may be a useful prognostic marker in ICU patients with pre-existing hypertension and diabetes, a subgroup that appears to be at particularly high risk of short-term mortality.

### 3.6. Cox Proportional Hazard Analysis

To further assess whether the association between high CAR and 30-day mortality persists after adjustment for potential confounders, we performed both univariable and multivariable Cox proportional hazards regression analyses. In the overall hypertensive cohort, high CAR was significantly associated with increased risk of 30-day mortality in both univariable analysis (HR: 3.02, 95% CI 1.48–6.17, *p* = 0.0024) and multivariable analysis (HR: 3.01, 95% CI 1.45–6.21, *p* = 0.0030) (Table 2).

In subgroup analyses, high CAR was also significantly associated with increased 30-day mortality among patients with normal/elevated BP in both univariable analysis (HR: 8.90, 95% CI: 2.00–39.17, *p* = 0.0038) and multivariable analysis (HR: 10.12, 95% CI: 2.20–46.59, *p* = 0.0030). In contrast, no statistically significant association was observed in stage 1 BP in either univariable analysis (HR: 1.78, 95% CI: 0.57–5.61, *p* = 0.3242) or multivariable analysis (HR: 2.70, 95% CI: 0.72–10.15, *p* = 0.1404), or in stage 2 BP in either univariable analysis (HR: 1.25, 95% CI: 0.28–5.60, *p* = 0.7682) or multivariable analysis (HR: 3.26, 95% CI: 0.37–29.19, *p* = 0.2901). In patients with both diabetes and normal/elevated BP, high CAR was significantly associated with increased 30-day mortality in both univariable analysis (HR: 10.00, 95% CI: 1.20–83.10, *p* = 0.0331) and multivariable analysis (HR: 19.41, 95% CI: 1.37–275.28, *p* = 0.0284) (Table 2).

## 4. Discussion

In this retrospective study of ICU patients with pre-existing hypertension, we found that higher CAR was associated with increased 30-day mortality in the overall hypertensive cohort, and this association remained significant after multivariable adjustment. The association was more pronounced in patients with normal/elevated BP, in whom CAR showed better discriminative performance and remained significantly associated with mortality in the multivariable Cox proportional hazard model analysis. Furthermore, in patients with diabetes and normal/elevated BP, CAR also showed a relatively high AUC and remained associated with increased 30-day mortality after adjustment. These results suggest that CAR, obtained after ICU admission, may serve as a useful prognostic marker in ICU patients with pre-existing hypertension, and more notably in those with hypertension combined with diabetes.

Several recent studies have investigated the prognostic role of CAR in patients with hypertension. In a large national cohort of hypertensive individuals, elevated CAR was independently associated with an increased risk of all-cause mortality (*n* = 9561; 48% male; mean age 57 ± 16 years; HR: 1.60, 95% CI: 1.23–2.09; *p* < 0.001) [16]. In a single-center retrospective study of hypertensive patients hospitalized with Coronavirus disease 2019 (COVID-19) and followed in the ICU, CAR was significantly higher in non-survivors, and a cutoff of 56.6 demonstrated good discriminative performance for predicting mortality (*n* = 281; 51.2% male; mean age 70.44 ± 11.08 years; HR: 4.909, 95% CI: 1.447–16.650; *p* < 0.001) [17]. A prospective study of patients with sudden sensorineural hearing loss found that those with hypertension had significantly higher CAR values and worse clinical profiles compared with those without hypertension (*n* = 120; 54.2% male; mean age 57.04 ± 14.15 (*p* = 0.014) [18]. In a large retrospective cohort of critically ill ICU patients, which included many individuals with hypertension, higher CAR at admission was an independent predictor of 30-day mortality (*n* = 6972; 62.6% male; mean age 64.9 ± 15.3 years; HR: 1.11, 95% CI: 1.09–1.14; *p* < 0.001) [19]. Similarly, in our study, CAR measured after ICU admission was significantly associated with increased 30-day mortality in overall hypertension patients (HR: 3.01, 95% CI 1.45–6.21, *p* = 0.0030) and those with normal/elevated BP (HR: 10.12, 95% CI 2.20–46.59, *p* = 0.0030), but not in those with stage 1, or stage 2 BP. To our knowledge, no prior work has examined CAR across different groups of hypertension.

Although CAR showed better prognostic performance and was significantly associated with a higher risk of 30-day mortality in patients with normal/elevated BP, but not in those with stage 1 or stage 2 BP, these seemingly counterintuitive results may have some possible explanations. In patients with stage 1 or stage 2 BP, the prognostic signal of CAR may be attenuated by a greater burden of chronic vascular injury, end-organ dysfunction, and multimorbidity. As a result, short-term mortality may be influenced by a broader set of competing risk factors, reducing the relative contribution of a single baseline inflammatory and nutritional marker such as CAR. In addition, patients with higher-stage hypertension may be more likely to receive chronic antihypertensive or cardioprotective therapies before ICU admission, which could alter inflammatory activity or modify the association between CAR and outcomes. It is also possible that in these groups, the acute severity and etiology of the critical illness at ICU presentation play a more dominant role in determining short-term mortality, thereby reducing the relative discriminatory value of CAR measured at baseline. Finally, the number of deaths in the stage 1 and stage 2 subgroups was limited, and the absence of significant associations in these subgroups may partially reflect reduced statistical power rather than a true lack of prognostic value. Future studies in larger ICU cohorts are needed to confirm whether these subgroup differences reflect true biological or clinical heterogeneity or are primarily driven by limited sample size.

In a retrospective cohort of 301 patients with diabetic foot infection, higher CAR was independently associated with increased risk of amputation, underscoring its clinical relevance in infection-related complications of diabetes [20]. Similarly, in patients with diabetic retinopathy, CAR values were significantly elevated compared with controls and closely correlated with disease severity, with ROC analysis demonstrating excellent discriminative accuracy [21]. Elevated CAR has also been reported in type 2 diabetes patients with diabetic nephropathy, where it independently predicted the presence of nephropathy and showed good diagnostic performance [22]. Moreover, in a cohort of 2755 diabetic patients undergoing percutaneous coronary intervention, higher CAR was associated with significantly worse five-year outcomes, including all-cause and cardiac mortality [23]. Arterial hypertension and diabetes mellitus frequently coexist and are linked by shared mechanisms, including chronic inflammation, endothelial dysfunction, vascular injury, and metabolic stress, all of which may worsen outcomes during critical illness [7]. In our study, CAR measured after ICU admission also demonstrated prognostic value in ICU patients with pre-existing hypertension and diabetes, a subgroup that appears to be at particularly high risk of short-term mortality. While previous studies have examined CAR in either hypertension or diabetes separately, the frequent coexistence of these conditions in clinical practice makes our analysis the first to evaluate CAR in this combined subgroup.

CAR, which combines CRP and albumin, reflects both systemic inflammation and nutritional status [24]. CRP rises rapidly after an inflammatory trigger and indicates the severity of the systemic response, whereas albumin decreases under stress, poor nutrition, and catabolism in critically ill individuals [25]. Together, these measures provide an integrated profile of a patient’s condition at ICU admission. However, both inflammation and nutritional status are dynamic during critical illness, and the prognostic value of CAR may evolve over time as the patient’s condition changes. Therefore, while the present study focused on the first available CAR measurement as an early baseline risk-stratification marker, CAR assessments during the first 48–72 h of ICU stay may provide additional prognostic information and should be examined in future studies.

In critically ill patients with hypertension, CAR may be elevated because CRP reflects vascular inflammation and endothelial dysfunction associated with long-standing hypertension, while hypoalbuminemia arises from endothelial leakage and impaired nutritional reserve, resulting in a higher ratio that signals poor prognosis. This pattern was even more pronounced in patients with diabetes, where chronic inflammation, endothelial injury, and low albumin are well-documented [7]. Although our study does not provide mechanistic evidence, these processes may help explain why CAR was strongly associated with 30-day mortality in this subgroup.

Our study has several strengths. First, we assessed CAR across different hypertension groups, providing insight that has not been specifically addressed in earlier research. Second, we examined its prognostic value in ICU patients with pre-existing hypertension and diabetes, a clinically relevant subgroup that has been largely overlooked despite the frequent coexistence of these conditions. Third, by focusing on CAR measured shortly after ICU admission and evaluating its association with 30-day mortality, our study highlights the potential utility of this marker for early risk stratification in critically ill patients. Nonetheless, several limitations should be acknowledged. Due to the retrospective nature of the study and the structure of the MIMIC-III database, the primary reason for ICU admission could not be determined with certainty from the available data. Moreover, blood pressure values at ICU admission may vary widely depending on the underlying acute illness and may not reflect chronic hypertension status. The retrospective design carries the risk of residual confounding. Other unmeasured factors, such as comorbidities or treatments, may have influenced the findings. Another limitation is that CAR was based on the first available CRP and albumin measurements after ICU admission. We did not evaluate the longitudinal changes in CAR during the first 48–72 h or throughout the ICU stay. As a result, the present findings reflect the prognostic value of early baseline CAR only, not how changes in CAR over time relate to outcomes. Future studies using longitudinal CAR measurements are needed to determine whether CAR trajectories offer additional real-time prognostic utility. In addition, the CAR thresholds were derived using the Youden index within the same study cohort and were not externally validated. Therefore, the cutoff values may reflect cohort-specific optimization and should not be interpreted as universally applicable thresholds. Subgroup analyses were also based on small numbers of events, particularly in stage 1 and stage 2 BP and diabetes. Thus, the corresponding effect estimates should be interpreted cautiously, as limited events may reduce precision. These results should therefore be considered exploratory and require confirmation in larger ICU cohorts. The AUC estimates from small subgroup analyses, particularly the diabetes with normal/elevated BP subgroup, should be interpreted cautiously because they were derived from limited event counts within the same dataset. Therefore, prospective evaluations and further validation are needed before these findings can be applied in clinical practice.

## 5. Conclusions

In this retrospective study of critically ill patients with pre-existing hypertension, CAR measured after ICU admission was associated with 30-day mortality in the overall hypertensive cohort. This association remained significant after multivariable adjustment and appeared more pronounced in patients with normal/elevated BP. In the subgroup of patients with diabetes and normal/elevated BP, CAR was also associated with increased 30-day mortality. These findings suggest that CAR may serve as a simple and practical tool for early risk stratification in this high-risk population, although prospective evaluations and external validations are needed before its routine application.

## Figures and Tables

**Table 1:** Baseline characteristics of ICU patients with pre-existing hypertension according to 30-day survival status.

Variable	Overall (*n* = 341)	Survivors (*n* = 306)	Non-Survivors (*n* = 35)	*p*-Value
**Demographic Characteristics**				
Gender				
Female, *n* (%)	148 (43.4)	132 (43.1)	16 (45.7)	0.911
Male, *n* (%)	193 (56.6)	174 (56.9)	19 (54.3)	
Age, years	65.17 [55.07–76.28]	64.12 [54.34–75.68]	72.00 [63.22–80.41]	0.011
**Comorbidities**				
Hypertension Group				
Normal/elevated BP *, *n* (%)	165 (48.38)	149 (48.7)	16 (45.71)	0.455
Stage 1 BP, *n* (%)	84 (24.7)	72 (23.6%)	12 (34.3)	
Stage 2 BP, *n* (%)	85 (25.0)	78 (25.6%)	7 (20.0)	
Hypertensive crisis, *n* (%)	7 (2.1)	7 (2.3)	0 (0.0)	
Diabetes, *n* (%)	113 (33.1)	100 (32.7)	13 (37.1)	0.576
**Laboratory/Clinical Variables**				
CRP, mg/L	56.40 [18.77–125.10]	52.70 [17.94–117.50]	89.90 [40.40–158.65]	0.012
Albumin, g/dL	2.96 (0.64)	2.98 (0.65)	2.78 (0.53)	0.038
CAR	19.54 [6.29–47.78]	18.09 [6.01–45.60]	35.48 [11.93–58.20]	0.008
WBC (×10^9^/L)	10.90 [7.70–14.80]	10.70 [7.57–14.80]	12.00 [9.05–15.00]	0.501
Creatinine, mg/dL	1.00 [0.70–1.30]	1.00 [0.70–1.30]	1.00 [0.80–1.40]	0.529

Abbreviation: ICU: intensive care unit, BP: blood pressure, CRP: C-reactive protein, CAR: C-reactive protein-to-albumin ratio, WBC: white blood cells; * BP was determined on ICU admission.

**Table 2:** Results of the univariate and multivariate Cox proportional hazard models showing the association between CAR and 30-day mortality in ICU patients with pre-existing hypertension and subgroups.

Population	Univariate Analysis			Multivariate Analysis		
	HR	95% CI	*p*-Value	HR	95% CI	*p*-Value
Overall hypertension	3.02	(1.48–6.17)	0.0024	3.01	(1.45–6.21)	0.0030
Normal/elevated BP *	8.90	(2.00–39.17)	0.0038	10.12	(2.20–46.59)	0.0030
Stage 1 BP	1.78	(0.57–5.61)	0.3242	2.70	(0.72–10.15)	0.1404
Stage 2 BP	1.25	(0.28–5.60)	0.7682	3.26	(0.37–29.19)	0.2901
Diabetes with Normal/elevated BP	10.00	(1.20–83.10)	0.0331	19.41	(1.37–275.28)	0.0284

Abbreviation: HR: hazard ratio, CI: confidence interval, BP: blood pressure. * BP was determined on ICU admission.

## Data Availability

The data used in this study are available from the MIMIC-III v1.4 database, which is publicly accessible to qualified researchers through PhysioNet after completing the required training and data use agreement. The filtered datasets generated and analyzed during this study are available from the corresponding author upon reasonable request.

## Supplementary Materials

The following supporting information can be downloaded at https://www.mdpi.com/article/10.3390/jcm15103683/s1. Table S1. International Classification of Diseases, Ninth Revision for hypertension and diabetes.

## Abbreviations

The following abbreviations are used in this manuscript:

CAR	C-reactive protein-to-albumin ratio
ICU	Intensive care unit
CRP	C-reactive protein
MIMIC III	Medical Information Mart for Intensive Care III
WBC	White blood cell
SD	Standard deviation
IQR	Interquartile range
ROC	Receiver operating characteristic
