# Supplementary material for: C-Reactive Protein-to-Albumin Ratio as a Prognostic Marker in ICU Patients with Pre-Existing Hypertension and Diabetes
Source: J Clin Med. 2026 May 11;15(10):3683. doi: 10.3390/jcm15103683 (PMC13206991; doi:10.3390/jcm15103683)
Supplement: Supplementary file 1 [file jcm-15-03683-s001.zip › jcm-4231067-supplementary.pdf]

Supplementary Materials

Table S1. International Classification of Diseases, Ninth Revision for hypertension and diabetes

**Condition**	**ICD-9 Codes**
Hypertension	401.0, 401.1, 401.9
Diabetes	250.xx

Abbreviations: ICD: International Classification of Diseases, Ninth Revision
